# Juggling Grandchild Care and Labor Force Participation: The Effect on Psychological Wellbeing of Older Women

**DOI:** 10.3389/fsoc.2021.806099

**Published:** 2022-01-19

**Authors:** Bruno Arpino, Daniela Bellani

**Affiliations:** ^1^ Department of Statistics, Computer Science, Applications, University of Florence, Firenze, Italy; ^2^ Department of Political and Social Sciences, Scuola Normale Superiore, Pisa, Italy

**Keywords:** grandchild care, labor force participation, work-family balance, psychological wellbeing, older adults

## Abstract

Although it is well-known that care responsibilities are strongly gendered also in later life, the consequences for older women of juggling work and care responsibilities are understudied. This study contributes to fill this gap by focusing on the wellbeing implications for older European women of combining work and grandchild care. The role strain and role enhancement theories guide our theoretical predictions. While the former predicts a lower wellbeing due to the double burden of grandchild care and paid work, the latter posits an increase in wellbeing through the accumulation of social identities or roles. By using longitudinal data from the Survey of Health, Ageing and Retirement in Europe (SHARE), we investigate whether grandmothers who do and those who do not work experience different levels of quality of life, depressive symptoms and life satisfaction. Our statistical model consists in a fixed-effect regression that adjusts for the lagged outcome. Results show that, among grandmothers engaged in paid work, grandchild care is not significantly associated with any of the three outcomes considered. Instead, non-working grandmothers seem to benefit from provision of grandchild care, in terms of higher quality of life and lower number of depressive symptoms. As thus, the provision of grandchild care tends to be beneficial for grandmothers’ wellbeing only if they do not combine this activity with paid work. Juggling paid work and childcare to grandchildren may result in an excessive burden which eliminates the potential benefits of grandchild care on older women’s wellbeing.

## Introduction

Work-family balance has been extensively studied among adult women. Research has widely documented the difficulties adult women face in juggling a career with care responsibilities toward their children or other relatives. Although it is well-known that care responsibilities are strongly gendered also in later life, the consequences of juggling work and caregiving are less investigated for older women. This study aims at contributing filling this gap by focusing on the role of combining work and grandchild care among older European women and on its implications for their wellbeing.

The literature on grandchild care has widely addressed the effects of the provision of care to grandchildren on health and wellbeing of grandparents. As highlighted in a recent systematic review (Danielsbacka et al., 2022), mixed evidence exists. Most cross-sectional studies on European countries reported positive associations between provision of grandchild care and grandparents’ wellbeing (e.g., Arpino et al., 2018 on life satisfaction; Danielsbacka and Tanskanen, 2016 on happiness). However, the majority of recent longitudinal studies find, instead, statistically insignificant associations (e.g., Ates, 2017 on self-rated health; Bordone and Arpino, 2019 on depression; Bordone and Arpino, 2021 on subjective age; Danielsbacka et al., 2019 on self-rated health, difficulties with activities of daily living, depressive symptoms, life satisfaction and meaning of life). Still, even among studies that attempt at estimating the causal effects of grandchild care provision, mixed findings have been reported with some studies documenting beneficial effects of caregiving. For example, studies employing instrumental variable techniques, have found positive effects on cognition (Arpino and Bordone, 2014) and mobility limitations (Ku et al., 2012).

In addition, previous research has identified positive effects for sub-groups of the population. The likelihood that grandchild care provision produces a positive impact on their wellbeing varies according to several factors, such as individual characteristics, caregiving situations (i.e., intensive and not intensive caregiving), social capital and normative context. One driving factor is associated with socio-demographic characteristics of grandparents. Studies have shown that higher educated grandparents have a higher ability, compared to their lower educated counterparts, of coping with stressful aspects of grandparenthood, thus preserving their wellbeing (e.g., Mahne and Huxhold 2015). Scholars have also identified women who became grandmothers for the first time and *via* their daughters (Di Gessa et al., 2020) as a group that experience a positive impact on wellbeing of becoming a grandmother. For what the intensity of caregiving is concerned, although studies on the overall relationship between the intensity of grandparental childcare and wellbeing do not come to a clear conclusion, scholars agree that grandfathers are usually negatively affected in terms of wellbeing by intensive care demands (e.g., Notter 2021). The level of social capital also contributes to define the boundaries of the wellbeing advantages/disadvantages of caring for grandchildren. In particular, research about intra-family strains and social supports has pointed out that closer familial relationships constitute a more relaxed living context for grandparents that is likely to lead to an improvement in their wellbeing (Sun and Dutta, 2021). Regarding the normative context, Arpino et al. (2018) found that, in Europe, it is in countries where intensive grandparental childcare is not common and less socially expected that grandparenthood has a stronger positive association with subjective wellbeing.

This strand of the literature, however, has overlooked the possible moderator role of paid work. This is unfortunate given that in today’s world people can expect to spend several years in the grandparent role during some of which they are still employed (Muller and Litwin, 2011). One strand of the literature has focused on the impact of becoming a grandparent on the employment status of older adults and, vice versa, on the impact of retirement on grandchild care provision. Rupert and Zanella (2018) found that, in the U.S., becoming a grandmother reduces women’s working time (of about 30%), while grandparenthood has no effect on men’s employment. Similar results are reported by Backhaus and Barslund (2021) and Zanasi et al. (2020) for the European context. Tanskanen et al. (2021), focusing on retirement transition, found a positive effect on grandchild care both for grandfathers and grandmothers.

A related strand of research has investigated the relationship between the provision of grandchild care and grandparents’ employment involvement. Lakomý and Kreidl (2015) shows that European grandmothers in part time positions are more likely to provide grand childcare compared to their full-time counterpart, but this is explained by the selection process into part-time/full-time contracts. Empirical evidence for overall Europe shows that, when selection is accounted for, even intensive grandchild care does not involve a conflictive relationship with employment for grandmothers (Floridi, 2020). According to Floridi (2020), this is especially the case in countries characterized by the so-called optional de-familisation regime–while in countries with familistic approaches to childcare the work-care conciliation appears to be more difficult. Zanasi et al. (2021) found that grandmothers in Italy were more likely to provide childcare if they worked during their adulthood as compared to grandmother who never worked, suggesting a role for work/family orientations and intergenerational transmission of work attachment.

Our research investigates whether the combination of paid work and grandchild care exerts an impact on grandparents’ wellbeing. This is crucial given that, as in the case of parents, on the one hand the double role of care provider and worker could negatively influence psychological conditions of grandparents (i.e., in terms of stress) (Kobayashi et al., 2013) but, on the other hand, it could promote positive attitudes towards life (Schmitz and Stroka 2013).

In order to explain the health and wellbeing consequences of family and other roles, previous studies have generally framed their contributions within the role enhancement and role strain theories (Moen et al., 1995). Role enhancement theory suggests that benefits may be gained by accumulation of social identities or roles (Sieber, 1974). This is consistent with the energy expansion perspective proposed by Marks (1977), that stresses that the provision of additional informal care provides higher individuals’ satisfaction. Roles provide access to social support and opportunities for gratification, and caregiving is considered a “productive role” (Rozario et al., 2004). Caregiving is viewed as one of the productive roles in which older people can contribute to the society and also improve one’s wellbeing (O’Reilly and Caro, 1995). On the same line of reasoning, Martire and Stephens (2003) speculate that individuals experience satisfaction in embodying multiple roles, because caring for the loved ones as well as being active in the labor market increase marginal utility. In this sense, various roles provide not only social and material gains but also an enhancement of resources such as self-esteem identity. Accordingly, these theoretical arguments predict positive outcomes also in terms of wellbeing for caregivers who are employed both in the labor market and in grandchildren caregiving.

The role strain theory, instead, argues that people have limited time, energy, and resources available to fulfil different social roles (Goode, 1960); thus, some of the older people still engaged in the labor market may find it difficult to fulfill the additional role of caregiver for grandchildren. In this sense, cross-life domains do not achieve a stable and favorable balance. On the contrary, work and care spheres create role pressure with negative consequences for other life dimensions, such as life satisfaction (e.g., Friedman and Greenhaus, 2000). Accordingly, given the time constraints due to the double-role, of worker and caregiver, grandparents could experience shortages in their leisure time sphere life (the so-called time-based conflict, Greenhaus and Beutell, 1985), thus influencing (indirectly) individual wellbeing (e.g., Chen et al., 2020). Role conflicts lead to stressful experiences and problematic interactions in the workplace sphere, too (Atienza and Stephens, 2000). When work-life conflict surges because of the strain felt in the care sphere difficulties to perform well in the workplace sphere can arise (the so-called strain-based conflict overcome; Greenhaus and Beutell, 1985). Life-work conflicts of grandparents may occur also when behaviors required in the role of caregiver are incompatible with behaviors required in the workplace. Grandparents experiencing behavioral strain, again, are supposed to be more likely to face a decline in their wellbeing. All these sorts of competition between the role of caregiving and worker are identified in the literature as competitive demand hypothesis (McMillan et al., 2011). All in all, embracing this perspective, grandparents who are engaged in both caregiving and work roles are expected to experience a lower level of wellbeing compared to their counterparts involved only in caregiving (or in paid work).

Research investigating the consequences of multiple role commitments among older adults is predominantly cross-sectional and focused on the roles of parent and partner. The combination of the grandparent role with other roles is under-investigated. The limited existing evidence on the effects of combining paid work and grandchild care is inconclusive. Some studies reported that working status moderates the influence of grandchild care obligations on health and wellbeing. For example, Szinovacz and Davey (2006) find that in the U.S. employment had a protective effect against potential negative effects of extensive grandchild care on depression. On the contrary, Chen and Liu (2012) show that in China even a low level of childcare involvement has a negative effect on health when is combined with paid work, suggesting the risk of double burden or role conflict. Meyer et al. (2012) reports mixed feelings associated with juggling work and grandchild care in the U.S.

Given that the theoretical arguments discussed above go in opposite directions and that the empirical evidence offers mixed results, we take an explanatory approach in examining the effect of the double burden of childcare provider and worker on psychological wellbeing of grandparents.

## Methods

### Data

We examine the effects on psychological wellbeing of combining paid work and grandchild care by using longitudinal data from the Survey of Health, Ageing and Retirement in Europe (SHARE; Börsch-Supan et al., 2013). SHARE is a longitudinal survey targeting individuals aged 50 and over and their partners in several European countries. We exclude wave 3 because it collected life histories and misses key variables of our interest. We also excluded wave eight because it was interrupted due to the COVID-19 pandemic.

We consider three outcome variables. First, we consider quality of life measured with the CASP-12 scale composed of four subscales, the initials of which make up the acronym: Control, Autonomy, Self-realization and Pleasure (CASP; the number 12 refers to the number of items). The 12 items which are presented as questions or statements to survey respondents are assessed on a four-point Likert scale (“often”, “sometimes”, “rarely”, “never”). The resulting score is the sum of these 12 items, and ranges from the minimum of 12 to the maximum of 48. The second dependent variable is number of depressive symptoms measured with the EURO-D scale, ranging from 0 to 12 (note that EURO-D is not an acronym; the name refers to the European Union initiative that proposed the scale; Prince et al., 1999). Finally, we examine life satisfaction, measured with the widely used Satisfaction with Life Scale: “On a scale from 0 to 10 where 0 means completely dissatisfied and 10 means completely satisfied, how satisfied are you with your life?”.

Our explanatory variables are dummy variables indicating whether the respondent is engaged or not in the provision of grandchild care and in paid work, respectively. Our main interest is on the interaction between the explanatory variables, and more specifically in the comparison of the effect on psychological wellbeing of grandchild care provision for women who do and those who do not work.

We control for several time-variant socio-demographic and health factors that may confound the relationship of interest, e.g. they may be associated with the explanatory variables and influence psychological wellbeing. Specifically, we account for age and its square (to allow for nonlinear effects), income, marital status (in a partnership—reference, never married, separated/divorced, widowed), number of grandchildren, distance in months between waves. As for income, we consider the total household income divided by the size of the household. The resulting values are finally divided by 10,000. We also control for two measures of health. First, we consider information on the experience of chronic diseases reported in response to the question, “Has a doctor ever told you that you had any of the following conditions: Hypertension, diabetes, cancer, lung disease, heart disease, stroke and arthritis?”. We include a dummy variable indicating whether the respondent reported at least one condition or not as a control. Second, we include a measure of activity limitations using the Global Activity Limitation Indicator (GALI). Thus, we add a binary variable taking value one for people who declared to be “limited, but not severely” or “severely limited” because of health in the activities people usually do, and 0 for respondents reporting not to be limited. Differences across countries in wellbeing are expected due, for example, to differences in welfare state provisions. However, as mentioned below, our fixed effects regression models remove time-invariant factors, such as country of residence and education.

Life satisfaction was measured differently in wave one and thus the regression analyses on this outcome excludes this wave. In addition, the three outcome variables presented a different number of missing values. Thus, the sample sizes slightly vary for the three outcomes. The number of women included in our regression analyses ranges between 29,226 (life satisfaction) and 32,868 (EURO-D), while the total number of observations ranges between 51,906 (life satisfaction) and 61,914 (EURO-D); see the bottom part of Table 2. The total number of observations multiplies the number of women included in our sample by the number of times they are observed. As a robustness check we implemented the analyses on the same sample obtained by dropping all missing values on the outcomes and results were very similar.

### Statistical Methods

We estimate panel data linear models with individual fixed effects. These models eliminate the influence of individual time-invariant observed and unobserved factors that might confound the relationship of interest. We use a dynamic version of the model where the lagged outcome is added among the control variables. In this way we account for possible reverse causality, i.e. for the possible effect of psychological wellbeing measured at the previous wave on subsequent work and caregiver statuses. We estimate two versions of each regression model without (M1) and with (M2) the inclusion of an interaction term between grandchild care and paid work.

## Results

We start by describing our sample. The top part of Table 1 reports descriptive statistics of all variables used in the regression analyses. The average age across all observations in our dataset was 69 (with a standard deviation of 9.8). Overall, considering all observations, 50% of grandmothers provided childcare to their grandchildren and 16% were engaged in paid work. The mean number of grandchildren was 3.1 (with a standard deviation of 3.2) and ranged between 0 and 27. This means that we included in our analyses grandchildless women that experienced the transition to grandparenthood during the period of observation.

**TABLE 1:** Descriptive statistics for all variables used in the regression models and transition rates for the explanatory variables.

Variable	Mean/%	Std. Dev	Min	Max
CASP	36.7	6.5	12	48
EURO-D	2.9	2.4	0	12
life satisfaction	7.5	1.9	0	10
provides grandchild care (%)	47.7	—	0	1
in paid work (%)	15.9	—	0	1
Age	69.0	9.8	50	101
in a partnership (%)	64.4	—	0	1
never married (%)	1.4	—	0	1
separated/divorced (%)	8.5	—	0	1
widowed (%)	25.7	—	0	1
illness (%)	56.0	—	0	1
GALI (%)	52.1	—	0	1
number of grandchildren	3.1	3.2	0	27
Income	2.0	4.6	0	711
distance between waves (months)	32.0	16.6	11	61
Transitions				
** **Time 1	Time 2			
	no care	care	no work	work
no care/no work	86.2	13.8	97.7	2.3
care/paid work	27.2	72.8	25.9	74.1

Time 1 and 2 represent a generic couple of consecutive waves.

The bottom part of Table 1 reports the conditional transition percentages for the two explanatory variables. More specifically, among those who did not provide care and those who did provide care we report the percentage of those who stayed in the same condition and those who changed their condition (first two columns of the bottom part of Table 2). Similarly, for not being in paid work/being in paid work (last two columns). Given that fixed effect models exploit within-individual variability over time to estimate the effects of interest, the transition probabilities are useful to assess whether enough changes are observed. About 14% of grandmothers who were not providing care at a given point in time (Time 1) provided care at a subsequent time (Time 2). Vice versa, 27% of caregivers were then found not to be engaged in grandchild care. For paid work, as expected given the age group we examine, almost all observed changes refer to working women that stopped working (26%).

**TABLE 2:** Estimated regression coefficients from fixed effects dynamic models for three measures of psychological wellbeing.

Independent variables	CASP		EURO-D		Life satisfaction	
	M1	M2	M1	M2	M1	M2
provides grandchild care	0.16**	0.21***	−0.05*	−0.08**	0.03	0.03
	(0.07)	(0.08)	(0.03)	(0.03)	(0.03)	(0.03)
in paid work	−0.08	0.06	−0.02	−0.10*	0.06	0.06
	(0.11)	(0.14)	(0.05)	(0.06)	(0.04)	(0.05)
grandchild care * work	—	−0.30*	—	0.17**	—	0.02
	—	(0.16)	—	(0.07)	—	(0.06)
Age	0.96***	0.97***	−0.27***	−0.27***	0.20***	0.20***
	(0.07)	(0.07)	(0.03)	(0.03)	(0.03)	(0.03)
age squared	−0.01***	−0.01***	0.00***	0.00***	−0.00***	−0.00***
	(0.00)	(0.00)	(0.00)	(0.00)	(0.00)	(0.00)
never married	0.15	0.14	−0.03	−0.02	−0.37	−0.37
	(0.78)	(0.78)	(0.35)	(0.35)	(0.27)	(0.27)
separated/divorced	−0.52	−0.52	0.26	0.26	−0.35**	−0.35**
	(0.39)	(0.39)	(0.16)	(0.16)	(0.15)	(0.15)
Widowed	−0.03	−0.03	0.73***	0.73***	−0.30***	−0.30***
	(0.15)	(0.15)	(0.06)	(0.06)	(0.06)	(0.06)
Illness	−0.41***	−0.41***	0.24***	0.24***	−0.09***	−0.09***
	(0.07)	(0.07)	(0.03)	(0.03)	(0.02)	(0.02)
GALI	−0.94***	−0.94***	0.46***	0.46***	−0.15***	−0.15***
	(0.07)	(0.07)	(0.03)	(0.03)	(0.02)	(0.02)
number of grandchildren	0.07***	0.07***	−0.02*	−0.02*	−0.00	−0.00
	(0.02)	(0.02)	(0.01)	(0.01)	(0.01)	(0.01)
Income	0.02**	0.02**	−0.01	−0.01	0.00	0.00
	(0.01)	(0.01)	(0.00)	(0.00)	(0.00)	(0.00)
distance btw waves	-0.01***	-0.01***	0.00***	0.00***	0.00***	0.00***
	(0.00)	(0.00)	(0.00)	(0.00)	(0.00)	(0.00)
lagged outcome	−0.20***	−0.20***	−0.21***	−0.21***	−0.33***	−0.33***
	(0.01)	(0.01)	(0.01)	(0.01)	(0.01)	(0.01)
Constant	14.50***	14.31***	11.32***	11.43***	2.25**	2.26**
	(2.37)	(2.37)	(0.88)	(0.88)	(1.12)	(1.12)
N (number of observations)	52766	52766	61914	61914	51906	51906
n (number of individuals)	29439	29439	32868	32868	29226	29226

Model 2 (M2) differs from model 1 (M1) only for the inclusion of the interaction between the provision of grandchild care and paid work. ***p < 0.01; **p < 0.05; *p < 0.1.

Table 2 reports the estimated coefficients from the regression models for the three outcome variables. Models that do not include the interaction between our explanatory variables (M1) show that women who provided grandchild care shown a statistically significantly higher quality of life (CASP) and lower number of depressive symptoms (EURO-D) compared to their non-caregiver counterparts. Instead, we do not find any statistically significant relationship between grandchild care provision and life satisfaction. Paid work was not significantly associated with any of the three measures of psychological wellbeing examined. Moving to the models that include the interaction term (M2), we found a statistically significant moderator effect of paid work for CASP and EURO-D. In both cases, the interaction term has an opposing sign to that of the coefficient of grandchild care, meaning that the beneficial effect of childcare on the two psychological wellbeing measures is reduced for working grandmothers. More specifically, given that the coefficient of the interaction is greater than that of grandchild care alone, the association between childcare provision and wellbeing even changes sign when care is combined with work. To better interpret the results, we estimated the Average Marginal Effect (AME) of grandchild care on CASP and EURO-D separately for women in paid work and those who are not engaged in paid work. Because we have a linear model, the AME of grandchild care on CASP for women who are not in paid work corresponds to the coefficient of grandchild care alone in M2 of Table 2 (AME = 0.21; *p* = 0.006). Due to the negative interaction term, the corresponding AME for women in paid work changes sign but it is not statistically significant (AME = −0.08; *p* = 0.585). We obtained a similar pattern of results for EURO-D (women not in paid work: AME = −0.08; *p* = 0.013; women in paid work: AME = 0.09; *p* = 0.163).

Although our interest was on the effect of combining grandchild care with paid work, it is worth noticing that paid work is negatively associated with depressive symptoms when not combined with grandchild care (M2, Table 2). It is also worth mentioning that the number of grandchildren is statistically significantly associated with higher quality of life and fewer depressive symptoms. Other controls also display a significant association with psychological wellbeing. As expected, worse health conditions are consistently associated with lower psychological wellbeing. The association between wellbeing and age follows an inverted U-shape patter. Income is positively associated with quality of life, while separated or divorced women tend to be less satisfied with their life compared to women in a partnership.

## Discussion

This paper contributed to two strands of the literature. On the one hand, the literature on work-family balance has widely documented the difficulties that adult women face in juggling a career with care responsibilities toward their children or other relatives. Although it is well-known that care responsibilities are strongly gendered also in later life, the consequences of juggling work and care are less known for older women. On the other hand, the consequences of grandchild care on grandparents’ health and wellbeing have been extensively studied. However, this research strand has rarely examined the effect of grandchild care when this activity is combined with other roles. This study aimed at contributing filling this gap by focusing on the role of combining grandchild care with paid work among older European women and on its implications for their psychological wellbeing.

Our analyzes relied on panel linear regression models with individual fixed effects and lagged outcomes estimated on data from the Survey of Health, Ageing and Retirement in Europe (SHARE). We considered three measures of psychological wellbeing: quality of life (CASP), depressive symptoms (EURO-D) and life satisfaction. Results suggest that grandmothers involved in childcare activities report a better quality of life and fewer depressive symptoms compared to older women who did not provide grandchild care. No statistically significant effect was detected, instead, on life satisfaction. When considering the interaction between grandchild care and paid work, the beneficial effect of grandchild care on quality of life and reduced depressive symptoms was confirmed only for non-working women. For women in paid work, instead, this beneficial effect of grandchild care provision was lost. In fact, no statistically significant association was detected within this group of older women between grandchild care provision and psychological wellbeing. Paid work was associated with fewer depressive symptoms among non-caregiver older women.

These results provide evidence in favor of both role enhancement and role strain theories. The role of childcare provider for grandmothers was found to produce certain positive effects on psychological wellbeing. A similar positive effect was found on depression for engagement in paid work. Although these effects were not established for all outcome variables we considered they confirm the prediction of role enhancement theories in that activation of roles may bring positive effects. However, our findings also demonstrated that when roles are combined, the positive effects of each role taken separately may be lost. This is particularly evident for depressive symptoms. For this outcome, in fact, empirical findings show that each of the two roles taken separately, i.e. grandchild care provision and paid work, implied reduced number of depressive symptoms as compared to the absence of these roles. Still, when the two roles are combined, these benefits are lost and older women who engaged simultaneously in grandchild care provision and paid work did not display significantly different levels of depression as compared to their counterparts who were not engaged in any of the two activities. This confirms the prediction of role strain theories that combination of different roles may become too stressful and burdensome. However, on a positive note, we did not find a determinantal effect per se of the roles’ combination compared to the absence of both roles, but that these two conditions were not significantly different.

The finding that grandchild care provision is more likely to display beneficial effects for grandmothers when this role is not combined with paid work is consistent with other studies on roles combination among grandmothers. A recent study (Arpino and Gómez-León, 2020), also based on SHARE data, found that older European women who provided grandchild care were less likely to be classified as depressed compared to their counterparts not involved in this childcare role. However, this beneficial effect of grandchild care vanished among grandchild care givers who were also engaged in other care activities (e.g., for the partner). Also in that case, taking on multiple roles was not found to be significantly worse in terms of psychological wellbeing than not being engaged in any care activity. However, taken together, these results represent a warning about the potential overload to which grandmothers can be exposed depending on other roles in which they may be active, being these paid work or other caregiving activities. An interesting avenue for future studies would be to examine the heterogeneity in the effects of combination of grandchild care provision with other roles depending on individual and contextual factors. At the individual level, for example, preferences may moderate the effect of multiple roles. A study focused on adults (Balbo and Arpino 2016), shown that the transition to motherhood had positive effects on subjective wellbeing only among family-oriented women. Similarly, among older women the effect of grandchild care, as well as that of its combination with paid work, could depend on family/work orientations. Other aspects, such as number of working hours, other caregiving roles, balance of care among partners should be deepened in future studies. Finally, the combined effect of caregiving and paid work in influencing wellbeing should be analyzed in future research in a cross-national perspective, accounting for differences across countries in policies, formal care services, labor market conditions and norms.

As mentioned in the Introduction, the literature on the effects of grandchild care on grandparents’ health and wellbeing offers mixed evidence. We found both evidence of a positive (for quality of life and depression) and of statistically insignificant (for life satisfaction) associations between grandchild care and psychological wellbeing. Previous research has shown that, when time-invariant unobserved confounders are accounted for through fixed effects model, the positive association between grandchild care and health/wellbeing often found in earlier studies disappears. Our results suggest that this is not always the case, and the effects of grandchild care may depend on the specific outcome considered. Future studies should investigate whether this is due to methodological issues (e.g., the scale of measurement of the different outcomes) or to substantive mechanisms.

This paper contributes to the literature on the consequences of grandchild care on grandparents’ health and wellbeing by focusing on the moderator role of paid work. Juggling paid work and childcare to grandchildren may result in an excessive burden, thus eliminating the potential positive effects of grandchild care provision on older women’s wellbeing.

## Data Availability

Publicly available datasets were analyzed in this study. This data can be found here: http://www.share-project.org/data-access.html.

## References

[B42] Arpino B. Bordone V. (2014). Does Grandparenting Pay Off? The Effect of Childcare on Grandparents’ Cognitive Functioning. J. Marriage Fam. 76, 337–351. 10.1111/jomf.12096

[B1] Arpino B. Bordone V. Balbo N. (2018). Grandparenting, Education and Subjective Well-Being of Older Europeans. Eur. J. Ageing 15 (3), 251–263. 10.1007/s10433-018-0467-2 30310372 PMC6156724

[B2] Arpino B. Gómez-León M. (2020). Consequences on Depression of Combining Grandparental Childcare with Other Caregiving Roles. Aging Ment. Health 24 (8), 1263–1270. 10.1080/13607863.2019.1584788 30870002

[B3] Ates M. (2017). Does Grandchild Care Influence Grandparents' Self-Rated Health? Evidence from a Fixed Effects Approach. Soc. Sci. Med. 190, 67–74. 10.1016/j.socscimed.2017.08.021 28843872

[B4] Atienza A. A. Stephens M. A. P. (2000). Social Interactions at Work and the Well-Being of Daughters Involved in Parent Care. J. Appl. Gerontol. 19 (3), 243–263. 10.1177/073346480001900301

[B5] Backhaus A. Barslund M. (2021). The Effect of Grandchildren on Grandparental Labor Supply: Evidence from Europe. Eur. Econ. Rev. 137, 103817. 10.1016/j.euroecorev.2021.103817

[B6] Balbo N. Arpino B. (2016). The Role of Family Orientations in Shaping the Effect of Fertility on Subjective Well-Being: A Propensity Score Matching Approach. Demography 53 (4), 955–978. 10.1007/s13524-016-0480-z 27306764

[B7] Bordone V. Arpino B. (2019). Grandparenthood, Grandparenting and Depression in 18 European Countries. J. Fam. Res. 31 (2), 216–239. 10.3224/zff.v31i2.06

[B8] Bordone V. Arpino B. (2021). Is There a Rejuvenating Effect of (Grand)childcare? A Longitudinal Study on German Data. J. Gerontol. B Psychol. Sci. Soc. Sci., gbab021. 10.1093/geronb/gbab021 33537773

[B9] Börsch-Supan A. Brandt M. Hunkler C. Kneip T. Korbmacher J. Malter F. (2013). Data Resource Profile: The Survey of Health, Ageing and Retirement in Europe (SHARE). Int. J. Epidemiol. 42, 992–1001. 10.1093/ije/dyt088 23778574 PMC3780997

[B10] Chen F. Liu G. (2012). The Health Implications of Grandparents Caring for Grandchildren in China. J. Gerontol. B Psychol. Sci. Soc. Sci. 67 (1), 99–112. 10.1093/geronb/gbr132 22156630 PMC3267025

[B11] Chen L. Fan H. Chu L. (2020). The Double-Burden Effect: Does the Combination of Informal Care and Work Cause Adverse Health Outcomes Among Females in China? J. Aging Health 32 (9), 1222–1232. 10.1177/0898264320910916 32248733

[B12] Danielsbacka M. Tanskanen A. O. Coall D. A. Jokela M. (2019). Grandparental Childcare, Health and Well-Being in Europe: A Within-Individual Investigation of Longitudinal Data. Soc. Sci. Med. 230, 194–203. 10.1016/j.socscimed.2019.03.031 31030010

[B13] Danielsbacka M. Křenková L. Tanskanen A. O. (2022). Grandparenting, Health, and well-Being: A Systematic Literature Review. Eur. J. Ageing, 19. 10.1007/s10433-021-00674-y PMC9424377 36052183

[B14] Danielsbacka M. Tanskanen A. O. (2016). The Association between Grandparental Investment and Grandparents' Happiness in Finland. Pers Relationship 23 (4), 787–800. 10.1111/pere.12160

[B15] Di Gessa G. Bordone V. Arpino B. (2020). Becoming a Grandparent and its Effect on Well-Being: The Role of Order of Transitions, Time, and Gender. J. Gerontol. B Psychol. Sci. Soc. Sci. 75 (10), 2250–2262. 10.1093/geronb/gbz135 31628843 PMC7664312

[B16] Floridi G. (2020). Daily Grandchild Care and Grandparents' Employment: A Comparison of Four European Child-Care Policy Regimes. Ageing Soc., 1–32. 10.1017/s0144686x20000987

[B17] Friedman S. D. Greenhaus J. H. (2000). Work and Family-Aallies or Enemies?: What Happens when Business Professionals Confront Life Choices. USA: Oxford University Press.

[B18] Goode W. J. (1960). A Theory of Role Strain. Am. sociological Rev. 25, 483–496. 10.2307/2092933

[B19] Greenhaus J. H. Beutell N. J. (1985). Sources of Conflict between Work and Family Roles. Acad. Manag. Rev. 10 (1), 76–88. 10.5465/amr.1985.4277352

[B20] Kobayashi M. Tamiya N. Kashiwagi M. Ito T. Yamaoka Y. Matsuzawa A. (2013). Factors Related to Positive Feelings of Caregivers Who Provide Home-Based Long-Term Care for Their Family Members in Japan. Res. Humanities Soc. Sci. 3 (16), 27–36. 10.7176/RHSS

[B21] Ku L.-J. E. Stearns S. C. Van Houtven C. H. Holmes G. M. (2012). The Health Effects of Caregiving by Grandparents in Taiwan: An Instrumental Variable Estimation. Rev. Econ. Household 10 (4), 521–540. 10.1007/s11150-012-9154-9

[B22] Lakomý M. Kreidl M. (2015). Full-time versus Part-Time Employment: Does it Influence Frequency of Grandparental Childcare? Eur. J. ageing 12 (4), 321–331. 10.1007/s10433-015-0349-9 28804364 PMC5549156

[B23] Mahne K. Huxhold O. (2015). Grandparenthood and Subjective Well-Being: Moderating Effects of Educational Level. J. Gerontol. B Psychol. Sci. Soc. Sci. 70 (5), 782–792. 10.1093/geronb/gbu147 25324294

[B24] Marks S. R. (1977). Multiple Roles and Role Strain: Some Notes on Human Energy, Time and Commitment. Am. sociological Rev. 42, 921–936. 10.2307/2094577

[B25] Martire L. M. Stephens M. A. P. (2003). Juggling Parent Care and Employment Responsibilities: The Dilemmas of Adult Daughter Caregivers in the Workforce. Sex Roles 48 (3), 167–173. 10.1023/a:1022407523039

[B26] McMillan H. S. Morris M. L. Atchley E. K. (2011). Constructs of the Work/Life Interface: A Synthesis of the Literature and Introduction of the Concept of Work/Life Harmony. Hum. Resource Develop. Rev. 10 (1), 6–25. 10.1177/1534484310384958

[B27] Meyer M. H. Timonen V. Arber S. (2012). “Grandmothers Juggling Work and Grandchildren in the United States,” in Contemporary Grandparenting. Changing Family Relationships in Global Contexts (Bristol, United Kingdom), 71–90. 10.1332/policypress/9781847429681.003.0004

[B28] Moen P. E. Elder G. H. Jr Lüscher K. E. (1995). Examining Lives in Context: Perspectives on the Ecology of Human Development. Washington, D.C.: American Psychological Association.

[B29] Muller Z. Litwin H. (2011). Grandparenting and Well-Being: How Important Is Grandparent-Role Centrality? Eur. J. Ageing 8 (2), 109–118. 10.1007/s10433-011-0185-5 25400536 PMC4231435

[B30] Notter I. R. (2021). Grandchild Care and Well-Being: Gender Differences in Mental Health Effects of Caregiving Grandparents. The Journals Gerontol. Ser. B, gbab164. 10.1093/geronb/gbab164 PMC9255931 34508596

[B31] O'Reilly P. Caro F. G. (1995). Productive Aging: An Overview of the Literature. J. Aging Soc. Pol. 6 (3), 39–71. 10.1300/j031v06n03_05 10186867

[B32] Prince M. J. Reischies F. Beekman A. T. Fuhrer R. Jonker C. Kivela S. L. (1999). Development of the EURO-D Scale-Aa European, Union Initiative to Compare Symptoms of Depression in 14 European Centres. Br. J. Psychiatry 174, 330–338. 10.1192/bjp.174.4.330 10533552

[B33] Rozario P. A. Morrow-Howell N. Hinterlong J. E. (2004). Role Enhancement or Role Strain. Res. Aging 26 (4), 413–428. 10.1177/0164027504264437

[B34] Rupert P. Zanella G. (2018). Grandchildren and Their Grandparents' Labor Supply. J. Public Econ. 159, 89–103. 10.1016/j.jpubeco.2017.12.013

[B35] Schmitz H. Stroka M. A. (2013). Health and the Double burden of Full-Time Work and Informal Care Provision - Evidence from Administrative Data. Labour Econ. 24, 305–322. 10.1016/j.labeco.2013.09.006

[B36] Sieber S. D. (1974). Toward a Theory of Role Accumulation. Am. sociological Rev. 39, 567–578. 10.2307/2094422

[B37] Sun K. Dutta M. (2021). Grandparenting in Rural China: A Culture-Centered Approach (CCA) to Understand Economic Inequality and Rural Labor Change. Qual. Soc. Work. 10.1177/14733250211018731

[B38] Szinovacz M. E. Davey A. (2006). Effects of Retirement and Grandchild Care on Depressive Symptoms. Int. J. Aging Hum. Dev. 62 (1), 1–20. 10.2190/8Q46-GJX4-M2VM-W60V 16454480

[B39] Tanskanen A. O. Danielsbacka M. Hämäläinen H. Solé-Auró A. (2021). Does Transition to Retirement Promote Grandchild Care? Evidence from Europe. Front. Psychol. 12, 738117. 10.3389/fpsyg.2021.738117 34616345 PMC8489495

[B40] Zanasi F. Sieben I. Uunk W. (2020). Work History, Economic Resources, and Women's Labour Market Withdrawal after the Birth of the First Grandchild. Eur. J. Ageing 17, 109–118. 10.1007/s10433-019-00525-x 32158376 PMC7040119

[B41] Zanasi F. Arpino B. Pirani E. Bordone V. (2021). Work Histories and Provision of Grandparental Childcare Among Italian Older Women. SocArXiv. 10.31235/osf.io/pdxfr

